# Duck Liver–associated Outbreak of Campylobacteriosis among Humans, United Kingdom, 2011

**DOI:** 10.3201/eid1908.121535

**Published:** 2013-08

**Authors:** Muhammad Abid, Helen Wimalarathna, Janette Mills, Luisa Saldana, Winnie Pang, Judith F. Richardson, Martin C. J. Maiden, Noel D. McCarthy

**Affiliations:** Public Health England, Chilton, UK (M. Abid, J. Mills, L. Saldana, J.F. Richardson, N.D. McCarthy);; University of Oxford, Oxford, UK (H. Wimalarathna, M.C.J. Maiden, N.D. McCarthy);; Reading Borough Council, Reading, UK (W. Pang)

**Keywords:** *Campylobacter*, *C. coli*, *C. jejuni*, campylobacteriosis, bacteria, poultry, duck, outbreak, enteric infections, United Kingdom, duck liver, human infection

## Abstract

*Campylobacter­* spp.–related gastroenteritis in diners at a catering college restaurant was associated with consumption of duck liver pâté. Population genetic analysis indicated that isolates from duck samples were typical of isolates from farmed poultry. *Campylobacter* spp. contamination of duck liver may present a hazard similar to the increasingly recognized contamination of chicken liver.

Although bacteria in the genus *Campylobacter* commonly cause gastroenteritis, identified outbreaks are relatively rare. In England and Wales, 21 identified campylobacteriosis outbreaks during 1992–1994 (*1*) and 50 during 1995–1999 (*2*) accounted for 0.2% and 0.4% of reported outbreaks of gastroenteritis, respectively. Water and milk were the main sources of *Campylobacter* spp. outbreaks in the United Kingdom and the United States, although becoming less so (*2*,*3*). Poultry consumption and restaurant dining are the most common foodborne illness risks, although many foodstuffs are implicated (*2*,*3*). Outbreaks associated with chicken liver pâté or parfait have increased: 14 outbreaks were associated with these items in England and Wales during 2007–2009 compared with 11 during the 15 preceding years (*4*). There were also large outbreaks in Scotland (*5*,*6*). The peer-reviewed literature identifies chicken as the type of poultry liver or refers to poultry without specifying type.

Multilocus sequence typing is increasingly used to identify animal origins of human campylobacteriosis (*7*). The presence of multiple *Campylobacter* strains (*6*) in individual outbreaks linked to chicken liver is consistent with documentation that chickens harbor multiple strains (*8*), that pâté is prepared from multiple livers (*5*,*6*), or both. We describe epidemiologic evidence for a duck liver pâté–associated outbreak and compare sequence types (STs) of isolates with animal and food isolate datasets.

## The Study 

The outbreak involved a group of 3 persons and a group of 29 persons who ate lunch at a catering college restaurant. A probable case-patient was defined as a restaurant diner with diarrhea onset within 7 days after eating at the restaurant on May 12, 2011. Infections were confirmed by laboratory test results.

Environmental health officers inspected the restaurant kitchen and reviewed food preparation processes on May 17. The lunches had been ordered in advance, and officers recorded the food choices made by each diner. Menu choices and occurrence of illness were verified by face-to-face interviews (22 diners), postal interviews (9 diners), and other diners for 1 diner who had died. When food consumption history differed from the diner’s lunch order, which occurred mainly through sharing of food, consumption history was used. Fisher exact test p-values and odds ratios with CIs were calculated for the association of each menu option with illness. All case-patients reported exposure to pâté. Lower CIs were estimated by using the Cornfield method in Stata 11 (StataCorp LP, College Station, TX, USA). Repeat analysis was restricted to patients with laboratory-confirmed illness and those who were not ill.

Symptomatic patients were requested to provide fecal samples. In addition, a sample of duck liver, not from the batch used to prepare the meals in question, was obtained from the supplier on June 13 and tested for *Campylobacter* spp. by using 25 g of sample cultured on Campylobacter Blood-Free Selective Agar Base after enrichment in Bolton broth (Oxoid, Basingstoke, UK). Multilocus sequence typing was performed by using standard methods. STs for samples from case-patients and the liver sample were compared with those of published isolates from chickens (mainly sampled in the United Kingdom during 2001–2005) (*9*,*10*), farmed ducks (sampled in the United Kingdom, 2007) (*11*), wild ducks (sampled in the United Kingdom, 2007) (*11*), and wild geese (sampled in the United Kingdom, 2002–2004) (*12*) by using a neighbor-joining algorithm and default parameters in MEGA (www.megasoftware.net/) as described (*13*).

**Figure 1:**
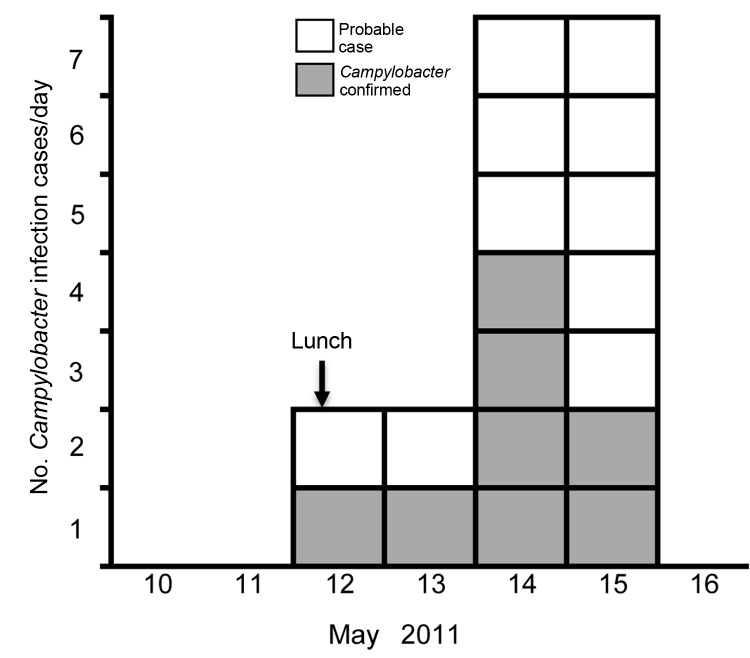
Onset dates of diarrheal illness related to a duck liver–associated outbreak of campylobacteriosis among humans, United Kingdom, 2011. Symptoms recorded with or without laboratory confirmation of *Campylobacter* infection, among persons eating lunch at a catering college restaurant on May 12, 2011. Vertical arrow indicates exposure date.

Of the 32 diners, 18 (56%) reported diarrhea: 8 had laboratory-confirmed campylobacteriosis, 6 had samples that were negative for *Campylobacter* infection, and 4 were not tested (Figure 1). Median duration of illness was 4 days; 1 case-patient died. Five case-patients described severe diarrhea (profuse, explosive, uncontrollable, or watery), 5 reported fever or shivering, and 2 reported abdominal pain. Consumption of duck liver pâté was strongly associated with illness. No other positive associations were identified (Table). When analysis was restricted to confirmed cases, campylobacteriosis was strongly associated with pâté (lower CI of odds ratio 5.5; p = 0.001).

**Table:** Association between food consumed and campylobacteriosis among diners at a catering college restaurant, United Kingdom, 2011

Food item	Foods diners ate			Foods diners did not eat		Attack rate, %	Odds ratio*	p value†
Symptomatic	Asymptomatic	Symptomatic	Asymptomatic
Starters								
Duck liver pâté	18	3		0	11	86	– (12.7–∞)	<0.001
Vegetable broth	2	11		16	3	15	0.030 (0.005–0.200)	<0.001
Main courses								
Pot roasted breast of lamb	12	9		6	5	57	1.1 (0.3–4.8)	1.00
Poached plaice in white wine sauce	5	5		13	9	50	0.7 (0.2–3.1)	0.71
Vegetarian polenta romaine	1	0		17	14	100	– (0.0-∞)	1.00
Desserts								
Vanilla gateaux chantilly	12	9		6	5	57	1.1 (0.3–4.8)	1.00
Chocolate pudding soufflé	5	5		13	9	50	0.7 (0.2–3.1)	0.71
Cheese	1	0		17	14	100	– (0.0-∞)	1.00

*95% Cornfield CIs are in parentheses. Where odds ratio is undefined, lower CI is presented. †By Fisher exact test.

Through review of cooking processes, we found that ≈1 kg of duck livers was seared and flambéed in batches without ensuring that adequate internal cooking temperatures were achieved. The seared livers were blended with other ingredients and chilled. No other high-risk ingredients or processes were identified. No illness among staff members was recorded on or immediately preceding May 12. A catering student who made and tasted the pâté became ill on May 16. No food samples remained.

**Figure 2:**
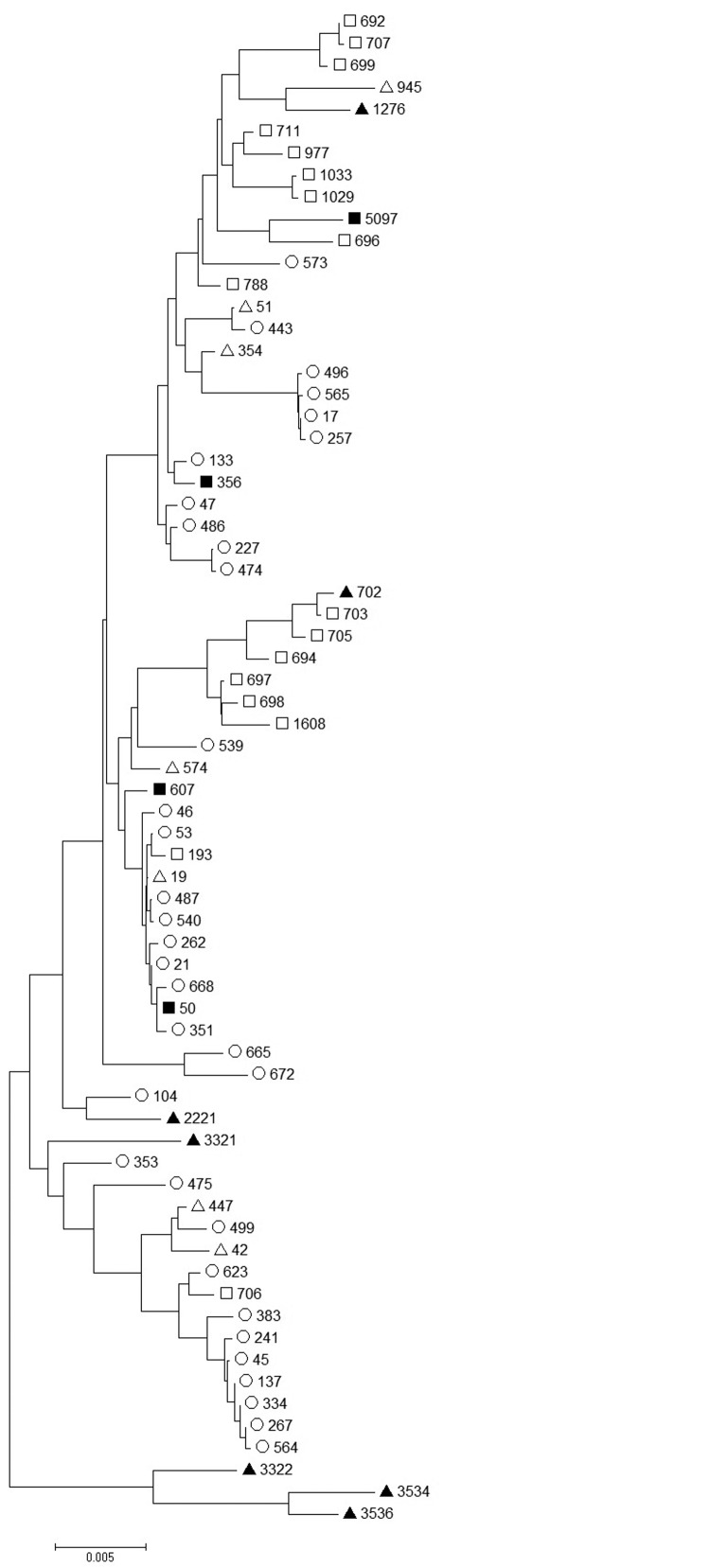
Comparison of *Campylobacter jejuni* sequence types (STs) from a duck liver–associated outbreak of campylobacteriosis among humans in the United Kingdom during 2011 (solid squares) with published sequence types of isolates from chicken (hollow circles) (*9**,**10*), domesticated duck (hollow triangles) (*11*), wild duck (solid triangles) (*11*), and wild geese (hollow squares) (*12*). ST5097 was isolated from a duck liver sample, ST356 from 3 case-patients, and ST50 and ST607 each from 1 case-patient. Scale bars indicates nucleotide substitutions per site.

*Campylobacter* isolates were available from 6 of 8 confirmed case-patients and the duck liver. One isolate was positive for *C. coli* and 5 for *C. jejuni*. The *C. jejuni* STs were ST356 (3 cases), ST50, and ST607. These STs are genetically diverse (Figure 2), but each clustered with chicken and farmed duck rather than wild waterfowl isolates. The duck liver isolate, ST5097, clustered with wild waterfowl isolates (Figure 2).

## Conclusions 

The attack rate of 86% among persons who ate duck liver pâté was similar to rates for outbreaks associated with chicken liver pâté (*5*,*6*). Pâté consumption was strongly associated with illness and laboratory-confirmed infection. Diners who did not eat this dish were unaffected. Pan frying of chicken livers is effective for killing internal *Campylobacter* spp. if the internal temperature reaches 70°C and is sustained for at least 2 minutes and if total cooking time is at least 5 minutes (*14*). The cooking process for the pâté, as reviewed by environmental health officers, was insufficient to kill bacteria inside the livers. This finding corroborates the epidemiologic evidence.

Aseptic testing of 30 chicken livers showed internal infection in 90% (*14*); testing of 50 chicken and 50 duck livers identified *Campylobacter* spp. contamination in 20 and 18, respectively (*15*). The high level of internal and external contamination in chicken liver in these studies and failure of insufficient cooking to destroy the bacteria in the current outbreak suggest that internal contamination of duck liver also occurs. Undercooked duck liver may therefore present a hazard similar to that presented by undercooked chicken liver. Cooking time should be sufficient to destroy bacteria throughout the liver. Deliberate undercooking was identified in 68% of 25 poultry liver–associated campylobacteriosis outbreaks that occurred during 1992–2009 (*4*). Outbreaks associated with chicken and duck liver pâté and parfait are being increasingly identified in the United Kingdom and are likely to occur in other countries because the cooking procedures described in the United Kingdom outbreaks are not based on recipes restricted to the United Kingdom. Sporadic cases associated with similar home cooking of poultry liver products are also likely to occur, but such cases will be difficult to identify unless specifically sought.

The diversity of isolates in this outbreak resembles that in an outbreak of campylobacteriosis related to chicken liver pâté (*6*). As with that outbreak, the diversity in the outbreak in this study could reflect individual livers co-infected with >1 *Campylobacter* strain, >1 infected liver in the food item, or both. This diversity suggests that bacterial invasion of chicken and duck livers is possible for a wide range of fairly distantly related *Campylobacter* spp. strains, including those of *C. jejuni* and *C. coli*. The clustering of *C. jejuni* isolates from this outbreak with STs associated with farmed duck and farmed chicken and the genetic separation from wild duck and wild goose isolates (Figure 2) suggests that the farm environment may favor some *Campylobacter* spp. subtypes sufficiently to overcome natural host associations. An alternative hypothesis is that among a wide range of subtypes infecting ducks, those that are found in other farm animals are more effective at causing human disease. The single *Campylobacter* isolate from a later, non–outbreak-associated batch of duck liver clustered with isolates from wild waterfowl rather than the outbreak isolates or other isolates from farmed ducks. The limited data on *Campylobacter* populations in poultry other than chickens restrict our ability to interpret this discrepancy. Further work to characterize the *Campylobacter* populations of wild and farmed ducks may facilitate more reliable inference.

## References

[1] Pebody RG, Ryan MJ, Wall PG. Outbreaks of *Campylobacter* infection: rare events for a common pathogen. Commun Dis Rep CDR Rev. 1997;7:R33–7 . 9080726

[2] Frost JA, Gillespie IA, O'Brien SJ. Public health implications of *Campylobacter* outbreaks in England and Wales, 1995–9: epidemiological and microbiological investigations. Epidemiol Infect. 2002;128:111–8. 10.1017/S0950268802006799 12002527 PMC2869802

[3] Friedman CR, Neimann J, Wegener HC, Tauxe RV. Epidemiology of *Campylobacter jejuni* infection in the United States and other industrialized nations. In: Nachamkin I, Blaser MJ, editors. *Campylobacter*. Washington (DC): ASM Press; 2000. p. 121–38.

[4] Little CL, Gormley FJ, Rawal N, Richardson JF. A recipe for disaster: outbreaks of campylobacteriosis associated with poultry liver pâté in England and Wales. Epidemiol Infect. 2010;138:1691–4. 10.1017/S0950268810001974 20727250

[5] O'Leary MC, Harding O, Fisher L, Cowden J. A continuous common-source outbreak of campylobacteriosis associated with changes to the preparation of chicken liver pâté. Epidemiol Infect. 2009;137:383–8. 10.1017/S0950268808001003 18647437

[6] Forbes KJ, Gormley FJ, Dallas JF, Labovitiadi O, MacRae M, Owen RJ, *Campylobacter* immunity and coinfection following a large outbreak in a farming community. J Clin Microbiol. 2009;47:111–6. 10.1128/JCM.01731-08 19005146 PMC2620832

[7] Sheppard SK, Dallas JF, Strachan NJ, Macrae M, McCarthy ND, Wilson DJ, *Campylobacter* genotyping to determine the source of human infection. Clin Infect Dis. 2009;48:1072–8. 10.1086/597402 19275496 PMC3988352

[8] Colles FM, McCarthy ND, Sheppard SK, Layton R, Maiden MC. Comparison of *Campylobacter* populations isolated from a free-range broiler flock before and after slaughter. Int J Food Microbiol. 2010;137:259–64. 10.1016/j.ijfoodmicro.2009.12.021 20071049 PMC3980632

[9] Manning G, Dowson CG, Bagnall MC, Ahmed IH, West M, Newell DG. Multilocus sequence typing for comparison of veterinary and human isolates of *Campylobacter jejuni.* Appl Environ Microbiol. 2003;69:6370–9. 10.1128/AEM.69.11.6370-6379.2003 14602588 PMC262249

[10] Sheppard SK, Dallas JF, MacRae M, McCarthy ND, Sproston EL, Gormley FJ, *Campylobacter* genotypes from food animals, environmental sources and clinical disease in Scotland 2005/6. Int J Food Microbiol. 2009;134:96–103. 10.1016/j.ijfoodmicro.2009.02.010 19269051 PMC3985063

[11] Colles FM, Ali JS, Sheppard SK, McCarthy ND, Maiden MCJ. *Campylobacter* populations in wild and domesticated Mallard ducks (*Anas platyrhynchos*). Environ Microbiol Rep. 2011;3:574–80. 10.1111/j.1758-2229.2011.00265.x PMC3229703 22164198

[12] Colles FM, Dingle KE, Cody AJ, Maiden MC. Comparison of *Campylobacter* populations in wild geese with those in starlings and free-range poultry on the same farm. Appl Environ Microbiol. 2008;74:3583–90. 10.1128/AEM.02491-07 18390684 PMC2423018

[13] Tamura K, Peterson D, Peterson N, Stecher G, Nei M, Kumar S. MEGA5: Molecular Evolutionary Genetics Analysis using maximum likelihood, evolutionary distance, and maximum parsimony methods. Mol Biol Evol. 2011;28:2731–9. 10.1093/molbev/msr121 21546353 PMC3203626

[14] Whyte R, Hudson JA, Graham C. *Campylobacter* in chicken livers and their destruction by pan frying. Lett Appl Microbiol. 2006;43:591–5. 10.1111/j.1472-765X.2006.02020.x 17083702

[15] Khalafalla FA. *Campylobacter jejuni* in poultry giblets [in German]. Zentralbl Veterinarmed B. 1990;37:31–4. 2346069 10.1111/j.1439-0450.1990.tb01023.x

